# Lipoprotein(a), an Opsonin, Enhances the Phagocytosis of Nontypeable *Haemophilus influenzae* by Macrophages

**DOI:** 10.1155/2021/2185568

**Published:** 2021-11-02

**Authors:** Zhi Liu, Yuxin Li, Yu Wang, Zhe Liu, Yan Su, Qiang Ma, Runlin Han

**Affiliations:** ^1^Research Center of Plasma Lipoprotein Immunology, Inner Mongolia Agricultural University, Hohhot, China; ^2^Laboratory of Biochemistry, Baotou Medical College, Baotou, China; ^3^Inner Mongolia Key Laboratory of Molecular Biology, Inner Mongolia Medical University, Hohhot, China; ^4^Hebei Changshan Biochemical Pharmaceutical Co., Ltd, Shijiazhuang, China

## Abstract

We recently showed that both nontypeable *Haemophilus influenzae* (NTHi) and its surface plasminogen- (Plg-) binding proteins interact with lipoprotein(a) (Lp(a)) in a lysine-dependent manner. Because Lp(a) can be taken up by macrophages, we postulated that it serves as an opsonin to enhance phagocytosis of NTHi by macrophages. Based on colony-forming unit (CFU) counts, Lp(a) was found to increase U937 macrophage-mediated phagocytosis of NTHi49247 and NTHi49766 by 34% and 43%, respectively, after 120 min. In contrast, Lp(a) did not enhance phagocytosis of *Escherichia coli* BL21 or *E. coli* JM109, which were unable to bind to Lp(a). As with U937 macrophages, Lp(a) was capable of increasing phagocytosis of NTHi49247 by peripheral blood mononuclear cell-derived macrophages. Opsonic phagocytosis by Lp(a) was inhibited by the addition of recombinant kringle IV type 10 (rKIV_10_), a lysine-binding competitor; moreover, Lp(a) did not increase phagocytosis of NTHi by U937 macrophages that were pretreated with a monoclonal antibody against the scavenger receptor CD36. Taken together, our observation suggests that Lp(a) might serve as a lysine-binding opsonin to assist macrophages in rapid recognition and phagocytosis of NTHi.

## 1. Introduction

Lipoprotein(a) (Lp(a)), discovered in 1963, is one of the enigmatic macromolecules in humans [1]. It has intrigued researchers owing to its highly polymorphic, ill-defined physiological function and association with atherosclerotic diseases [2]. Lp(a) has a complex structure consisting of one low-density lipoprotein- (LDL-) like core that is covalently attached by a disulfide bond to a unique glycoprotein apolipoprotein(a) (apo(a)) [3]. The LDL-like moiety of Lp(a) is essentially indistinguishable from LDL with regard to the presence of apolipoprotein B-100 (apoB100) and its lipid composition [4]. Apo(a) shares extensive amino acid homology with plasminogen (Plg), an important serine protease zymogen in the fibrinolytic system, and contains multiple copies of a Plg kringle IV- (KIV-) like domain, followed by a Plg kringle V- (KV-) like domain and an inactive protease domain [5]. Unlike Plg, there are ten subtypes of kringle IV (KIV_1_–KIV_10_) domains in apo(a), each of which is present in one copy, with the exception of KIV_2_ that appears in different repeat numbers, accounting for the high heterogeneity of apo(a) [6]. As the domain most closely resembling the Plg kringle IV domain, apo(a) KIV_10_ contains a strong lysine-binding site (LBS), which is thought to bind to various biological substrates such as fibrin, cell surface receptors, and extracellular matrix (ECM) proteins, via lysine-dependent mechanisms [7]. Numerous studies have revealed that Lp(a)/apo(a) may inhibit the binding or activation of Plg and ultimately interfere with fibrinolysis [8].

*Haemophilus influenzae* is a human-specific gram-negative coccobacillus that requires hemin and NAD for growth [9]. Nontypeable *H. influenzae* (NTHi) is a nonencapsulated, commensal microbe in the respiratory tract and a common cause of mucosal infections such as otitis media, sinusitis, conjunctivitis, exacerbations of chronic obstructive pulmonary disease (COPD), and cystic fibrosis (CF) [10]. NTHi has developed an array of strategies to facilitate the infection and invasion of hosts. For instance, Plg recruitment is one of the most important strategies employed by NTHi for invasion as it facilitates adhesion to tissues, evasion of the immune response, and penetration of the ECM [11]. NTHi surface-associated proteins, including protein E (PE) [12], protein F (PF) [13], and aspartase [14], have been identified as Plg-binding proteins that participate in Plg capture. In addition, both intact NTHi and its primary Plg-binding protein, PE, were demonstrated to bind to Plg via lysine residues [12].

Plg recruitment is commonly exploited by many infectious agents [15], which employ surface Plg-binding proteins to hijack the proteolytic activity of plasmin(ogen) and degrade tissue barriers for further dissemination [16]. The interaction between infectious agents and Plg is often lysine-dependent [17]. In 2010, Han suggested that Lp(a) may play a role in host defense by inhibiting Plg recruitment in response to pathogens [18]. Recently, our laboratory investigated the interactions between Lp(a) and Plg-binding proteins of NTHi and found that recombinant PE [19], PF [13], and aspartase [20] bind to Lp(a) through its carboxy-terminal lysine residues. In addition, Lp(a) particles were more readily taken up by macrophages [21, 22]. Based on this evidence, we proposed that Lp(a) may serve as an opsonin to mediate the phagocytosis of NTHi by macrophages.

In this study, we sought to determine whether Lp(a) exhibits opsonic activity to facilitate macrophage phagocytosis and, if so, to identify the binding mechanism involved in this function.

## 2. Materials and Methods

This study was approved by the Life Sciences Department of the Inner Mongolia Agricultural University. Participation was voluntary, and written informed consent was obtained from each participant for the use of blood samples in this research.

### 2.1. Isolation and Purification of Lp(a)

A strong anion exchanger column was previously reported [23] to have excellent resolution for LDL and Lp(a), with detection of as little as 1% LDL. Therefore, we coupled one-step density gradient ultracentrifugation with anion exchanger chromatography to obtain the pure form of Lp(a). Fasting venous blood from a healthy donor was collected to obtain plasma by centrifugation at 1800 × *g* for 10 min. 4 mL of plasma, which was adjusted to a density of 1.21 g/mL, was introduced into the bottom of an ultracentrifuge tube for 4 mL. KBr solutions in concentrations of 1.1 g/mL (2 mL), 1.063 g/mL (3 mL), and 1.006 g/mL (1 mL) were overlaid sequentially on the plasma to form a discontinuous gradient. After centrifugation at 207,099 × *g* and 10°C for 3 h, the resulting layer (*d* = 1.063 g/mL), containing a mixture of Lp(a) and LDL, was separated and dialyzed for 24 h against 20 mM Tris-HCl (pH 7.4) containing 150 mM NaCl and 1 mM EDTA at 10°C. Lp(a)-rich fractions were further loaded onto a HiTrap QFF column attached to an ÄKTA Avant 25 system (GE Healthcare, Danderyd, Sweden). The column was eluted with 300 mM NaCl, 20 mM Tris, and 1 mM EDTA (pH 7.4). Protein concentrations of Lp(a) were determined by using the BCA Protein Assay Kit (Pierce, Rockford, IL, USA). And the absence of endotoxin was verified using the LAL Chromogenic Endotoxin Quantitation Kit (Pierce).

The integrity and purity of Lp(a) were assessed by SDS-PAGE and western blotting. Lp(a) was probed with a polyclonal anti-apo(a) antibody (1 : 20,000, Fitzgerald Industries, Acton, MA, USA) and a polyclonal anti-apoB100 antibody (1 : 20,000, Sigma-Aldrich, St. Louis, MO USA), followed by a corresponding HRP-conjugated secondary antibody (1 : 5000, R&D Systems, Minneapolis, MN, USA). Signal detection was performed with enhanced chemiluminescence (Tiangen Biotech Co., Ltd., Beijing, China) by using G:Box Chemi XT4 (Syngene, Cambridge, UK). Lp(a) was stored in 1.5 mL airtight sterile vials at 4°C.

### 2.2. Purification of rKIV_10_

For protein expression, the transformant *E. coli* BL21/pASK-IBA37-rKIV_10_ was cultivated overnight in 30 mL of LB medium containing 100 *μ*g/mL ampicillin at 37°C on an orbital shaker at 200 rpm. Thereafter, the bacteria culture (2 mL) was inoculated in 200 mL LB medium with 100 *μ*g/mL ampicillin and incubated at 37°C with agitation at 200 rpm until OD reached 0.6. Then, expression was induced by the addition of 0.2 *μ*g/mL of anhydrotetracycline followed by 3 h further incubation using the same conditions. Cells were harvested and suspended in lysis buffer (137 mM NaCl, 2.7 mM KCl, 1.6 mM Na_2_HPO_4_, 147 mM KH_2_PO_4_, and 5 mM EDTA, pH 8.0).

The bacterial cells were lysed by one freeze-thaw cycle and digested with DNase, PMSF, and lysozyme. After washing, the lysate was dissolved in 7 M urea (pH 8.0) containing 50 mM *β*-mercaptoethanol, 20 mM Tris-HCl, 0.5 M NaCl, and 5 mM imidazole with constant shaking for 1 h. The supernatant was passed through 0.45 *μ*m filters (Millipore, Bedford, MA, USA) and diluted 2.5-fold with binding buffer (0.5 M NaCl, 50 mM Tris-HCl, 7 M urea, and 5 mM imidazole, pH 8.0). Then, the solution was applied to Ni Sepharose 6 Fast Flow resin (GE Healthcare, Buckinghamshire, UK) containing 0.5 mL resin. After treatment with wash buffer (0.5 M NaCl, 50 mM Tris-HCl, 7 M urea, and 30 mM imidazole, pH 7.4), rKIV_10_ was eluted with 5 mL of denaturing elution buffer (0.5 M NaCl, 50 mM Tris-HCl, 7 M urea, and 300 mM imidazole, pH 7.4). The flow through was refolded using dialysis solution (20 mM Tris-HCl containing 150 mM NaCl and 1 mM EDTA, pH 7.4) supplemented with oxidized and reduced glutathione (1.25 mM each) at 4°C for 18 h before being dialyzed against dialysis solution for 24 h. Purity of the protein was assessed by SDS-PAGE stained with Coomassie blue. Endotoxin in rKIV_10_ was removed with High-Capacity Endotoxin Removal Resin (Pierce) up to an endotoxin content of less than 0.1 EU/mL.

### 2.3. Bacterial Strains and Cultures

Standard laboratory strains NTHi49247 and NTHi49766 were acquired from the American Type Culture Collection (ATCC) (Manassas, VA). Both NTHi strains were grown for 21 h at 37°C in a humid atmosphere containing 5% CO_2_ on brain heart infusion- (BHI-) agar plates supplemented with 10 *μ*g/mL hemin and 10 *μ*g/mL NAD (sBHI). NTHi cultures were inoculated into fresh sBHI broth and shaken at 200 rpm and 37°C until the cells reached the midlog phase (OD_600_ = 0.70). *E. coli* BL21 (DE3) and JM109 (Sangon Biotech, Shanghai, China) were grown on LB agar plates overnight at 37°C. *E. coli* colonies were harvested and resuspended in 50 mL fresh LB broth for subculture up to the midlog phase (OD_600_ = 0.70), while shaking at 200 rpm and 37°C.

### 2.4. Enzyme-Linked Immunosorbent Assay (ELISA)

Intact bacteria (10^8^/well) resuspended in PBS (137 mM NaCl, 3 mM KCl, 8 mM Na_2_HPO_4_, and 1.5 mM KH_2_PO_4_, pH 7.4) were coated on 96-well flat bottom microplate wells (Greiner Bio-One, Frickenhausen, Germany) by incubation at 25°C for 1.5 h. Redundant bacterial cells were removed by TBST, and the plate was blocked with 200 *μ*L of 1% BSA in TBST for 1.5 h. Lp(a) or LDL (increasing concentrations 10-fold from 0.05 *μ*g protein/mL to 5 *μ*g/mL) in TBST at 100 *μ*L was added to each well for 1.5 h. In competitive ELISA, increasing concentration of the competitor ligand was added to the binding reactions and allowed to bind for 1.5 h at 25°C. The polyclonal anti-apo(a) antibody (1 : 4,000, Fitzgerald) or polyclonal anti-LDL antibody (1 : 10,000, Sigma-Aldrich) was used for capture, and the HRP-conjugated donkey anti-goat antibody (1 : 1,000, R&D Systems) was used for detection. A washing buffer with 0.5% Tween 20 in TBS was used between steps, each for 3 times. Plates were developed with a tetramethylbenzidine substrate (TMB, Promega, Madison, WI, USA) and read at 450 nm in a microplate reader (Synergy HT, BioTek Instruments Inc., VT, USA).

### 2.5. Bacterial Adherence Assays

Bacterial adherence assays were performed as previously described [24] with slight modifications. 6-well plates with 100 *μ*g Lp(a) or BSA were incubated for 2 h at 25°C. After two washes with Dulbecco's PBS (DPBS), midlog phase bacterial cells were added and incubated for 2 h at 25°C. Thereafter, unbound bacteria were removed by washing whereas the bound bacteria were gram-stained. Wells were mounted with a coverslip and examined by using an Olympus BX41 microscope at 400x amplification.

### 2.6. U937 Cells

The human monocytic leukemia cell line U937 (CRL-1593.2; ATCC) was propagated in complete RPMI 1640 (Gibco, CA, USA) supplemented with 10% FBS (Gibco, Victoria, Australia), 2 mM glutamine, and 100 units/mL penicillin/streptomycin solution in an atmosphere of 5% CO_2_ at 37°C. To induce differentiation, U937 cells in suspension (5.0–5.5 × 10^5^ cells per mL) were differentiated by incubation with phorbol myristate acetate (PMA; 160 nM; Sigma-Aldrich) for 48 h. Cell Counting Kit-8 (CCK-8) was used to evaluate the densities and the activity of cells. In some experiments, cells were pretreated with anti-CD36 mAb (SAB4700166, Sigma-Aldrich) at a final concentration of 7 *μ*g/mL for 3 h following 45 h of PMA stimulation. An irrelevant anti-CD4 mAb (SAB4700054, Sigma-Aldrich) was used as a negative control. Redundant antibodies were washed by corresponding medium coincubation.

### 2.7. Peripheral Blood Mononuclear Cell-Derived Macrophages

Peripheral blood mononuclear cells (PBMCs) were obtained from a healthy donor using Ficoll-Paque Plus (GE Healthcare, Uppsala, Sweden), according to the manufacturer's instructions. After several steps of washing with RPMI 1640 medium and removal of nonadherent cells, the remaining PBMCs were cultured overnight with complete RPMI 1640 at 37°C in 5% CO_2_. Thereafter, PBMCs were collected and plated in 12-well plates at 3 × 10^5^ cells per well. Cells were differentiated following the same protocol with U937 cells.

### 2.8. Phagocytosis Assay

Before phagocytosis assays, the adhering cell sheet was washed with prewarmed DPBS and then replaced with fresh medium. Bacteria suspended in RPMI 1640 were added to a 6-well plate concurrently with or without specific proteins. After a 120 or 160 min coincubation at 37°C in 5% CO_2_, bacterial cells were harvested and then serially diluted and plated onto agar to determine the number of CFUs. Besides, quantities of viable cells were also determined by relative fluorescence intensities (RFI). 300 *μ*L of the sample was pelleted and resuspended in 130 *μ*L DPBS. Bacteria were labeled with 10 *μ*g/mL Hoechst 33342 (Sigma-Aldrich) on ice for 5 min in the absence of light, and fluorescence was assessed by fluorometric analysis (Synergy HT, BioTek Instruments Inc.) using an excitation wavelength of 360 nm and emission wavelength of 460 nm. The proteins used in this study, namely, Lp(a), LDL, BSA, and/or rKIV_10_, were added at a concentration of 10 *μ*g/mL unless otherwise stated. For the experiments with NTHi, RPMI 1640 was supplemented with hemin and NAD, both at a concentration of 10 *μ*g/mL. In addition, 10% sBHI broth was also used to replenish all solutions to maintain the vigor of NTHi cells. All phagocytosis assays were performed in sterile medium, in the absence of antibiotics and endotoxin. Each condition was performed in triplicate, in three independent experiments, for each bacterial strain.

To examine intracellular bacterial internalization by U937 macrophages, we used a modification of the method as described previously [25]. Briefly, the nonadhered bacteria after incubation with macrophages were removed by washing thrice with sPBS followed by using 0.25% Trypsin-Versene to detach the cells. All cells were mechanically lysed by vigorous vortexing for 1 min. To determine the number of viable intracellular bacteria, the lysates were serially diluted for 10,000 times and plated on sBHI agar plates. Colony-forming units were counted after 24 h of incubation at 37°C.

For the evaluation of phagocytosis efficiency, each treatment was estimated by two subgroups, bacteria only and treatment of bacteria with macrophages. The phagocytosis was calculated with the following formula to assess phagocytosis efficiency: (*A* − *B*)/*A*, where *A* is the value of bacteria with or without treatment after the indicated incubation period in culture and *B* is the value of bacteria with the same treatment and incubation period as that of *A* but with coincubation with macrophages. When it comes to change of phagocytosis, the results were obtained by subtraction of the number of the lipoprotein-free control group. The applicable multiplicity of infection (MOI) values for NTHi49247, NTHi49766, *E. coli* BL21, and *E. coli* JM109 were 50 : 1, 314 : 1, 6 : 1, and 25 : 1, respectively.

### 2.9. Fluorescence Microscopy

After the phagocytosis assay, bacteria and cells in 6-well plates were separated for staining in the preparation for fluorescence microscopy. Bacteria from each well were collected and stained with 200 *μ*g/mL FITC solution (10 mg/mL DMSO, Sigma-Aldrich) in DPBS for 30 min at 37°C and 5% CO_2_, followed by two DPBS washes. Cells in 6-well plates were gently washed with DPBS and then stained with 1.25 *μ*g/mL DAPI (4′,6-diamidino-2-phenylindole, Sigma-Aldrich) for 5 min at room temperature, followed by a one-step wash. The stained bacteria were added to macrophages and a coverslip mounted over the wells and then analyzed using fluorescence microscopy (Model BX41, Olympus, Japan). The images were merged using Image-Pro Plus 6.0.

### 2.10. Statistical Analysis

Data are expressed as means ± standard deviation (SD) and representative of a minimum of three independent experiments conducted in triplicate. Statistical analysis was performed using two-way ANOVA with the Bonferroni post hoc test for comparison of three or more groups. All statistical tests were performed using Prism version 5.0 (GraphPad Prism 8), and a value of *P* < 0.05 was considered statistically significant.

## 3. Results

### 3.1. Isolation and Purification of Lp(a)

**Figure 1:**
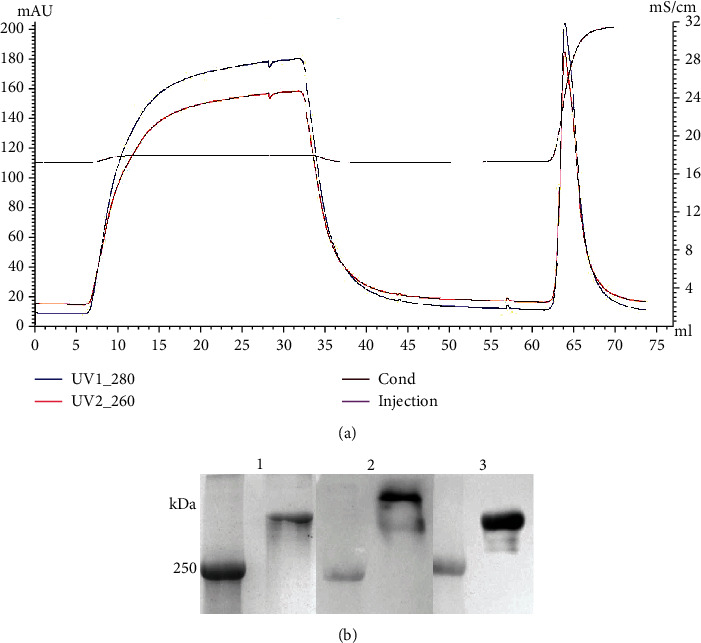
Purification of Lp(a). (a) Elution profile of Lp(a). A mixture of Lp(a) and LDL was run through a Q FF strong anion exchanger column and eluted with 300 mM NaCl, 20 mM Tris, and 1 mM EDTA (pH 7.4). The second peak corresponds to Lp(a). (b) SDS-PAGE and immunoblot analyses of Lp(a). SDS-PAGE gel stained with Coomassie blue (lane 1); an identical gel subjected to western blotting with either anti-apoB100 Ab (lane 2) or anti-apo(a) Ab (lane 3).

To isolate highly purified Lp(a), we coupled one-step density ultracentrifugation with anion exchanger chromatography. First, we obtained a lipoprotein fraction of 1.063 containing a mixture of Lp(a) and LDL. Lp(a) was subsequently separated by anion exchanger chromatography, resulting in one clear Lp(a) peak, as shown in Figure 1(a). The electrophoretic banding pattern and results of western immunoblot analysis, using anti-apo(a) and anti-apoB100 antibodies for reduced Lp(a), are shown in Figure 1(b). The results indicated that the same sample of Lp(a) could react with either anti-apo(a) or anti-apoB100 antibody. Coomassie blue staining of gels showed only one band, at the position corresponding to apoB100.

### 3.2. Interactions between Lp(a) and Bacteria

**Figure 2:**
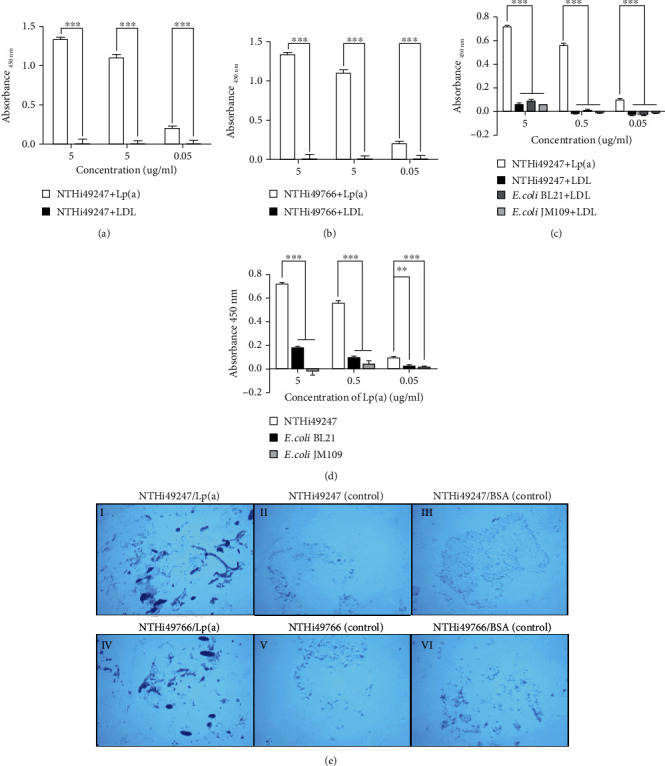
Interactions between Lp(a) and bacteria were analyzed by ELISA and bacterial adherence assay. (a–d) Binding of Lp(a) to bacteria was analyzed by whole-cell ELISA. Purified Lp(a) or LDL was added to intact bacteria at increasing concentrations (0.05–5 *μ*g/mL). The bound fraction of Lp(a) was measured using anti-apo(a) Ab, and that of LDL was measured using anti-LDL Ab. Lp(a) bound to each of the two strains of NTHi in a dose-dependent manner, whereas the negative control protein LDL did not (a, b). Two strains of *E. coli* showed weak binding to both Lp(a) and LDL (c, d). Data shown are mean values from three experiments conducted in duplicate, and error bars indicate SDs. Statistical significance was determined by two-way ANOVA and the Bonferroni post hoc test. ^∗^*P* < 0.05, ^∗∗^*P* < 0.01, and ^∗∗∗^*P* < 0.001. (e) Attachment of NTHi to immobilized Lp(a) was visualized by light microscopy after standard gram staining. Wells, either coated or uncoated with BSA, were included as negative controls. Representative images of two independent experiments are shown.

Binding of purified Lp(a) to two strains of NTHi (NTHi49247 and NTHi49766) was assessed by whole-cell ELISA, with LDL being the negative control. Bacteria were incubated with Lp(a) or LDL, at different concentrations, for binding and subsequent detection with their specific antibodies. Lp(a) bound to the two strains of NTHi in a dose-dependent manner, whereas LDL showed almost no ability to bind to either strain (Figures 2(a) and 2(b)). Two strains of *E. coli* (*E. coli* BL21 and JM109) were also subjected to whole-cell ELISA. Data showed that Lp(a) bound only weakly to *E. coli* BL21 (Figure 2(c)) and did not bind to *E. coli* JM109 at all (Figure 2(c)). Similarly, there was almost no detectable binding between LDL and the two strains of *E. coli* (Figure 2(d)). Therefore, *E. coli* BL21 and JM109 were chosen as Lp(a) nonbinding controls for phagocytosis assays.

Interactions between the two strains of NTHi were confirmed by bacterial adherence assays. To observe the attachment of bacteria to immobilized Lp(a), we applied the two strains of NTHi to six-well plates coated with Lp(a). Both the NTHi strains were found to adhere to the Lp(a)-coated wells (Figure 2(e) I and IV), whereas they showed only slight adherence to blank wells (Figure 2(e) II and V) and BSA-coated wells (Figure 2(e) III and VI), which were included as an additional negative control.

### 3.3. Lp(a) Opsonized NTHi for Phagocytosis by U937 Macrophages

To evaluate the effects of Lp(a) on phagocytosis, we first determined the applicable infection ratio of different bacteria after 40 and 80 min coincubation by CFU counts. Macrophages added with bacteria showing positive phagocytosis were considered the suitable multiplicity of infection (MOI) (data not shown). The time course of uptake was further examined every 40 min until 160 min of incubation, and it was found that 120 minutes was an appropriate initial time to examine the effect of Lp(a). Phagocytosis efficiency of the protein-free control group was used as the benchmark to calculate the relative phagocytosis efficiency in CFU or RFI on the addition of other ligands.

**Figure 3:**
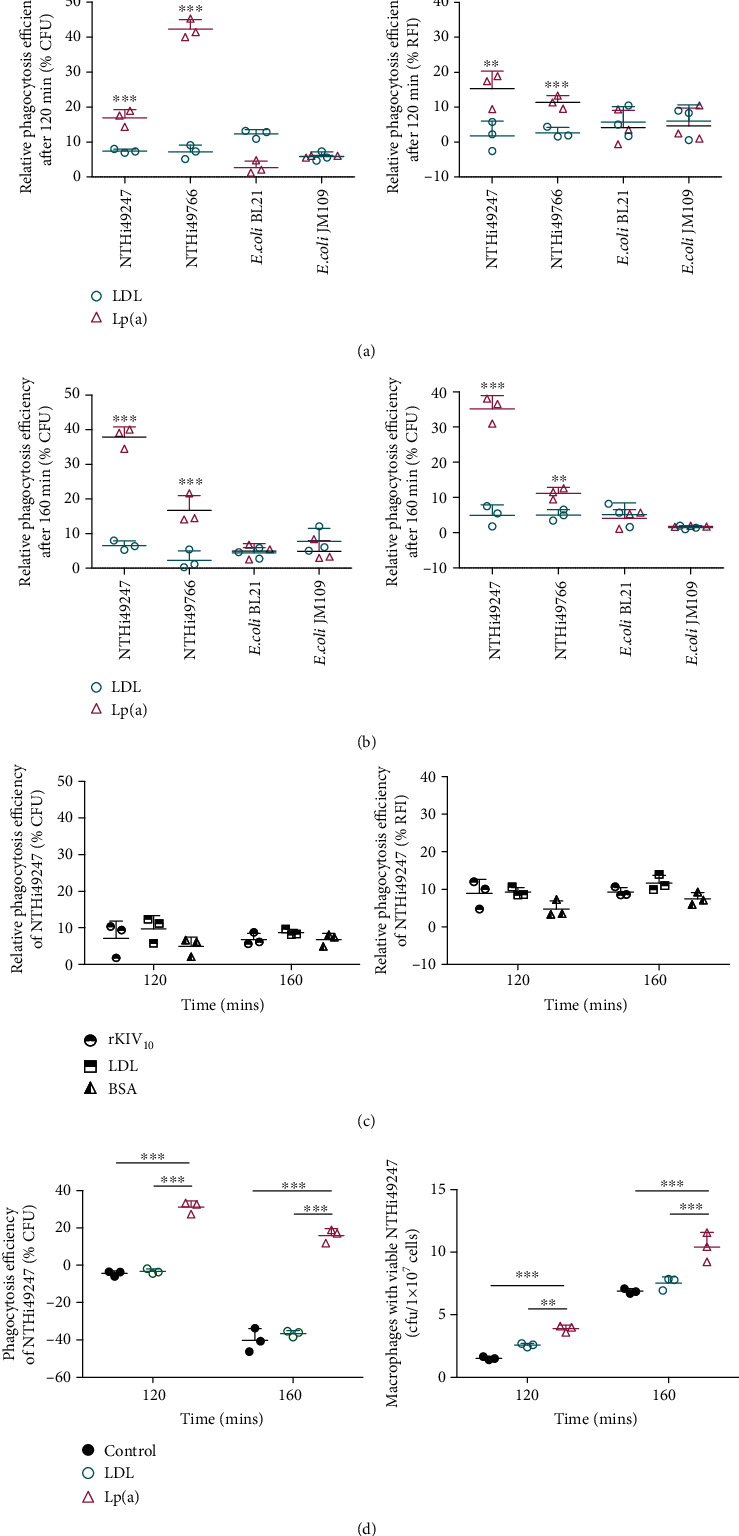
Lp(a) opsonized NTHi for phagocytosis by U937 macrophages. (a, b) The phagocytosis of NTHi49247, NTHi49766, *E.coli* BL21, and *E.coli* JM109 by U937 macrophages was estimated by CFU counts and RFI measurements after a (a) 120 or (b) 160 min incubation. Efficiency of phagocytosis is indicated with the following formula: (*A* − *B*)/*A*, where *A* is the value of bacteria with or without treatment after the indicated incubation period in culture and *B* is the value of bacteria with the same treatment and incubation period as that of *A* but with coincubation with macrophages. The relative phagocytosis efficiency of protein-treated bacteria was calculated by using the protein-free control group in the same experiment as a benchmark. Raw fluorescence measurements were corrected using a buffer blank. Each experiment was conducted at least in triplicate. Data are shown as means ± SD. Statistical significance of samples with bacteria+Lp(a) versus bacteria+LDL was determined by two-way ANOVA with the Bonferroni post hoc test (^∗∗^*P* < 0.01, ^∗∗∗^*P* < 0.001). (a, b) The CFU and RFI values at two time points of the macrophages phagocyting Lp(a)-treated NTHi49247 and NTHi49766 were significantly higher than those of the cells phagocyting LDL-treated microbes. And for two strains of *E.coli*, there was no significant difference in CFU and RFI values of the cells engulfing Lp(a)-treated and LDL-treated microbes. (c) Change of phagocytosis efficiency of NTHi49247 in the presence of rKIV_10_, LDL, BSA. No significant differences among those three treatments. (d) Lp(a) promotes the internalization of NTHi49247 into U937 macrophages. Along with the significant increased phagocytosis of NTHi49247-Lp(a) by U937 macrophages, the intracellular viable NTHi49247 in U937 macrophages is significantly increased. Statistical significance of samples with bacteria+Lp(a) or LDL versus bacteria alone was calculated by two-way ANOVA with the Bonferroni post hoc test (^∗∗^*P* < 0.01, ^∗∗∗^*P* < 0.001).

The phagocytic efficiency of Lp(a)-treated NTHi49247 calculated by CFU showed an increase by 17% and 38% compared to that of the lipoprotein-free control at 120 and 160 min, respectively (Figures 3(a) and 3(b)). This efficiency was 10% and 31% higher, respectively, than that in the LDL-treated group. For NTHi49766, Lp(a) also enhanced phagocytic efficiency by 42% and 17% compared to the control group at 120 and 160 min, respectively (Figures 3(a) and 3(b)). Compared to the LDL-treated group, phagocytosis was promoted by 35% and 14% (Figures 3(a) and 3(b)). The result revealed that the efficiency of both NTHi49247 and NTHi49766 uptake by U937 macrophages was significantly enhanced by the presence of Lp(a) in comparison to LDL. To further estimate that the specific binding between Lp(a) and NTHi was the chief cause of phagocytosis enhancement, we further estimated the effect of Lp(a) on phagocytosis of two strains of nonbinding bacteria, *E. coli* BL21 and JM109. Compared to that of the control group, similar results were observed with Lp(a) and LDL in phagocytosis of both *E. coli* BL21 and JM109 (Figures 3(a) and 3(b)).

Besides, we noticed that LDL and Lp(a) could also promote phagocytosis of nonbinding bacteria to varying degrees (Figures 3(a) and 3(b)). To further explore the phenomenon, BSA and rKIV_10_ were employed as negative controls in a phagocytosis assay with LDL. Compared to the control group, similar increases (approximately less than 10% increase compared to the protein-free control group) in phagocytosis efficiency were observed in LDL-, rKIV_10_-, and BSA-treated groups (Figure 3(c)). The results indicated that promotion of phagocytosis could be induced by different types of proteins without a binding ability to bacteria.

To verify that the higher reduction of extracellular bacteria of the Lp(a) treatment group was major due to opsonization, we further determined the number of intracellular NTHi after 120 or 160 min incubation. With significant reduction of extracellular Lp(a)-treated NTHi49247, the internalization of bacteria, respectively, increased by 158% and 51% compared to the control group, whereas the corresponding enhancement compared to that of the LDL-treated group was 52% and 38%, respectively. In all cases, RFI measurements showed similar trends (Figures 3(a)–3(d)).

Cumulatively, the increase in phagocytosis of NTHi induced by Lp(a) compared to the LDL group likely results from the binding ability of NTHi to Lp(a), in addition to nonspecific effects. These observations suggest that Lp(a) is the primary factor responsible for the observed increase in phagocytosis of NTHi by U937 macrophages.

### 3.4. rKIV_10_ Inhibited Lp(a)-Mediated Phagocytosis

**Figure 4:**
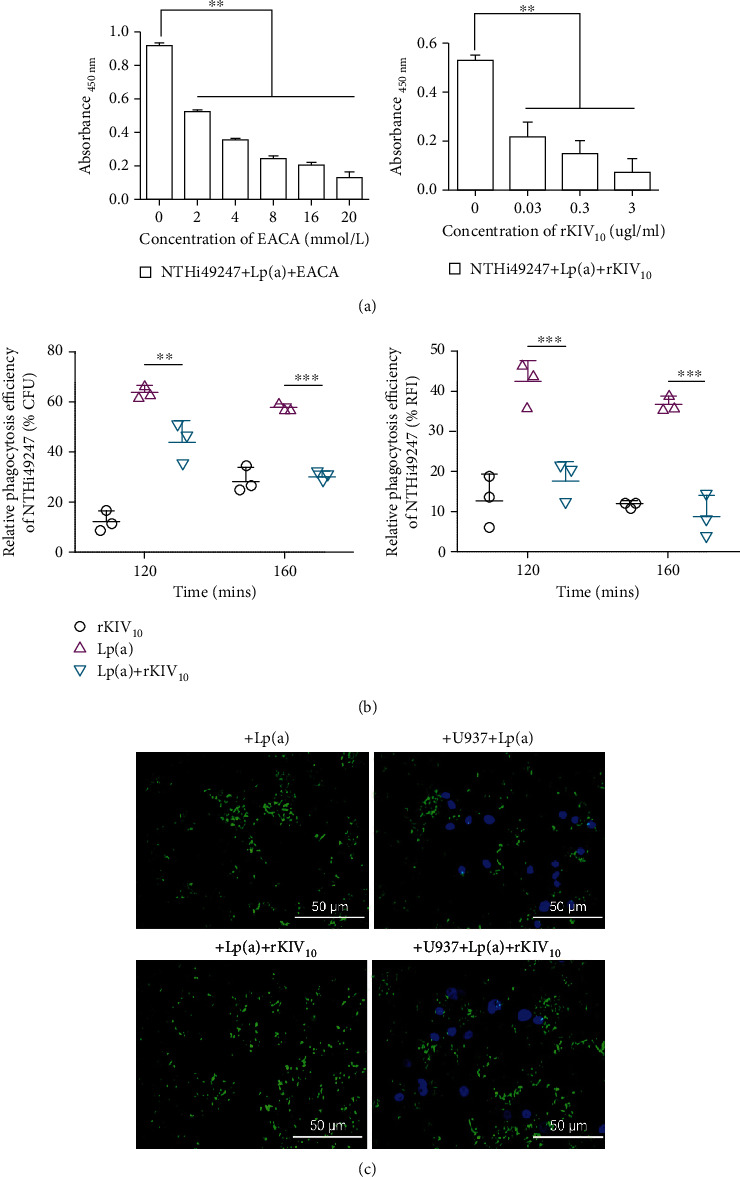
Increase in response to Lp(a) was largely inhibited by the addition of rKIV_10_. Whether the interaction between NTHi and Lp(a) was lysine-dependent was determined by whole-cell ELISA. The mixture of Lp(a) and different concentrations of EACA (0-20 mM) and rKIV_10_ (0.03-3 *μ*g/mL) was used. (a) The binding of Lp(a) to intact NTHi was inhibited by two inhibitors in a dose-dependent manner. Statistical significance was determined by two-way ANOVA and the Bonferroni post hoc test. (b) Inhibition of Lp(a)-mediated phagocytosis of NTHi49247 by rKIV_10_ was estimated by CFU counts and RFI measurements. (c) Representative photographs of rKIV_10_- or Lp(a)-treated FITC-NTHi49247 (green) incubated with or without macrophages stained with DAPI (blue). The MOI of NTHi49247 was 56 : 1. Data are represented as means ± SD from three independent experiments. Assays were conducted on at least three separate occasions. Statistical significance of the differences between groups in the phagocytosis assay was determined by two-way ANOVA and the Bonferroni post hoc test. ^∗∗^*P* < 0.01, ^∗∗∗^*P* < 0.001.

Considering that NTHi has been previously shown to bind to Plg-binding proteins via lysine residues, we sought to determine if the same is true for NTHi binding of Lp(a). To this end, EACA, a lysine analog, and rKIV_10_, the canonical LBS in Lp(a), were utilized to explore their binding interactions via ELISA. Results show that binding between NTHi and Lp(a) was inhibited by both EACA and rKIV_10_ in a dose-dependent manner, confirming our assumption (Figure 4(a)). To further examine the interaction between Lp(a) and NTHi in phagocytosis, we applied recombinant KIV_10_ to the Lp(a)-mediated phagocytosis assay with NTHi49247 and found that compared to that of the protein-free control, phagocytosis efficiency of the Lp(a)-treated group was significantly increased by 63% and 57% at 120 and 160 min according to CFU counts (Figure 4(b)), whereas this effect was largely inhibited by the addition of rKIV_10_. The addition of rKIV_10_ at the same time as Lp(a) resulted in 19% and 30% decrease in opsonization at each time point according to CFU counts (Figure 4(b)). RFI measurements and fluorescence micrographs showed similar results (Figures 4(b) and 4(c)). These observations indicated that the phagocytosis increase in NTHi caused by Lp(a) may be inhibited by rKIV_10_, the construct containing a strong lysine-binding ability. And the inhibition only partially but not entirely may be due to the nonspecific promotion of rKIV_10_ in phagocytosis. Collectively, the interaction in phagocytosis between Lp(a) and NTHi was lysine-dependent. Lp(a) through its LBSs promotes the phagocytosis of NTHi by macrophages.

### 3.5. Anti-CD36 Antibody Abolished Opsonization by Lp(a)

**Figure 5:**
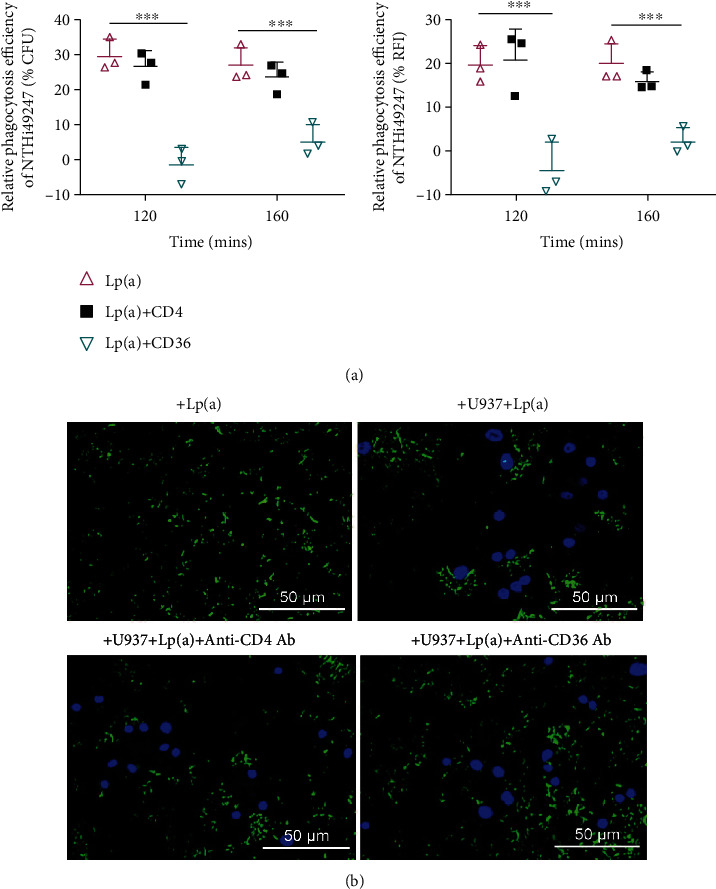
Treatment with an anti-CD36 antibody abrogated Lp(a)-mediated phagocytosis. (a) Phagocytosis efficiency was determined by CFU counts, RFI values, and (b) fluorescence micrographs. The MOI of NTHi49247 was 68 : 1. The study was repeated three times, with each treatment performed in triplicate. Data are shown as means ± SD. Statistical significance of the differences between groups was estimated using two-way ANOVA and the Bonferroni post hoc test. ^∗∗∗^*P* < 0.001.

After determining the binding pattern between Lp(a) and NTHi, we further investigated the macrophage receptors involved in Lp(a)-mediated opsonization. Anti-CD36 mAb and an irrelevant anti-CD4 mAb (negative control) were used in the Lp(a)-mediated phagocytosis assay. Treatment with anti-CD36 mAb or anti-CD4 mAb did not observably alter phagocytosis of NTHi49247 from that of the untreated group (data not shown). Further, the significant enhancement of phagocytosis induced by Lp(a) was abrogated at 120 min following administration of the anti-CD36 antibody. CFU counts also decreased by 30% and 22%, respectively, at 120 and 160 min, as did RFI measurements and fluorescence micrographs (Figures 5(a) and 5(b)). In comparison, treatment with anti-CD4 mAb resulted in almost no change in the ability of Lp(a) to mediate phagocytosis of NTHi (Figures 5(a) and 5(b)). These data indicated that the scavenger receptor CD36 on macrophages was primarily responsible for recognizing the Lp(a)-bacteria complex and participating in opsonic phagocytosis mediated by Lp(a).

### 3.6. Lp(a) Enhanced Phagocytosis of NTHi by U937 Macrophages in a Dose-Dependent Manner

**Figure 6:**
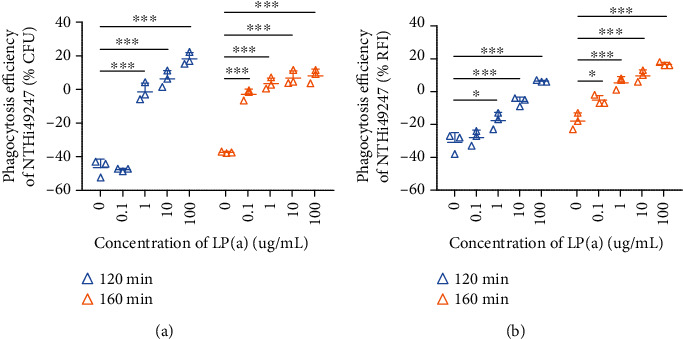
Lp(a) enhanced phagocytic efficiency of U937 macrophages in a dose-dependent manner. Lp(a) at 10-fold increasing doses, from 0.1 *μ*g/mL to 100 *μ*g/mL, was used in the phagocytosis assay, and its effect was evaluated by CFU counts (a) and RFI values (b). The MOI of NTHi49247 was 63 : 1. Values represent means ± SD from three independent experiments. Statistical significance of the differences between groups was determined by two-way ANOVA and the Bonferroni post hoc test. ^∗^*P* < 0.05, ^∗∗∗^*P* < 0.001.

Concentrations of Lp(a) in plasma vary up to 1000-fold, ranging from less than 0.1 mg/dL to over 100 mg/dL, across the human population [2, 26]. The average plasma concentration of Lp(a) is reportedly 10 mg/dL [27]. To verify the opsonization by Lp(a), we chose an Lp(a) concentration range over four orders of magnitude (10-fold increasing doses from 0.1 *μ*g/mL to 100 *μ*g/mL) to assess the effects of the protein on phagocytosis. CFU counts indicated that Lp(a), at concentrations of 1, 10, and 100 *μ*g/mL, significantly increased phagocytosis efficiency at 120 min by approximately 45%, 53%, and 65%, respectively. At 160 min, Lp(a) at each concentration showed a dose-dependent increase in phagocytosis efficiency (Figure 6(a)). Comparable trends were observed in RFI values (Figure 6(b)).

### 3.7. Lp(a) Enhanced Phagocytosis of NTHi by PBMC-Derived Macrophages

**Figure 7:**
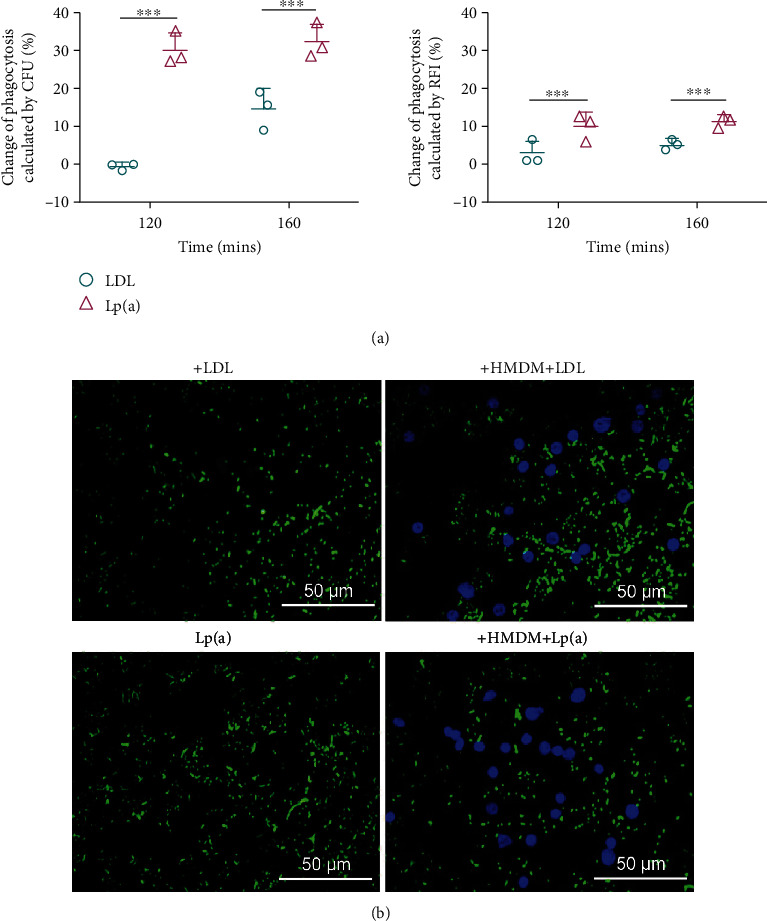
Lp(a) enhanced PBMC-derived macrophage phagocytosis of NTHi. The effects of Lp(a) on PBMC-derived macrophage phagocytosis of NTHi were determined by (a) CFU counts, RFI measurements, and (b) fluorescence micrographs. The MOI of NTHi49247 was 60 : 1. Data are shown as the means ± SD from three independent experiments. Statistical significance of the differences between groups was determined by two-way ANOVA with the Bonferroni post hoc test. ^∗∗∗^*P* < 0.001.

To confirm the effect of Lp(a) on opsonization, we repeated the NTHi assays, substituting HMDMs for the U937 macrophages. PBMCs, isolated from a healthy donor and differentiated with PMA, were used in the phagocytosis assays. Based on CFU counts, the phagocytic efficiency of HMDMs in the Lp(a)-treated group increased by 30% and 33% at two time points compared to that in the lipoprotein-free control and by 29% and 18% compared to that in the LDL-treated group (Figures 7(a) and 7(b)). The RFI values mirrored the CFU counts. These results suggested that Lp(a) could also serve as an opsonin in phagocytosis of NTHi by HMDMs.

## 4. Discussion

In this study, we identified a previously unreported role for Lp(a) in innate immunity, namely, opsonization in macrophage phagocytosis. Lp(a) can act as an opsonin to induce more efficient phagocytosis of NTHi by both U937 and PBMC-derived macrophages, through lysine-dependent binding of Lp(a) to NTHi. The *in vitro* phagocytosis model in the study was aimed at establishing a natural system to investigate the spontaneous process in phagocytosis. As the interaction among proteins, bacteria, and macrophages is complex, the assessments containing CFU counts, RFI measurements, and fluorescent micrographs were used to reveal a more complete phagocytosis state. To isolate the opsonic activity of Lp(a) from other possible protein-cell interactions that may be cooccurring, LDL was introduced as a control that does not bind to bacteria. *E. coli* BL21 and JM109, neither of which binds to Lp(a) or LDL, were also employed as controls in the study. Of note, we observed that Lp(a) caused a remarkably greater increase in phagocytosis of NTHi than did LDL because bacteria have an affinity for binding Lp(a) but not for LDL. Alternatively, in the cases of nonlipoprotein-binding *E. coli*, the improved phagocytosis efficiency induced by Lp(a) was less than or equal to that of LDL. Hence, Lp(a) was suggested to act as an opsonin to aid in the rapid recognition and uptake of NTHi by phagocytes. We further determined the viable NTHi inside U937-derived macrophages after phagocytosis. During the same time of experiments, the treatment of Lp(a) induced more number of viable NTHi than LDL. It is clear from the results that Lp(a) is involved in enhancing the uptake of NTHi, but its influence on intracellular killing is not entirely clear because NTHi could survive in macrophages, as reported previously [25]. In addition, the fact that opsonization by Lp(a) was largely inhibited by the strong lysine-binding competitor rKIV_10_ indicated the effect to be dependent on lysine-binding. Thus, phagocytosis mediated by Lp(a) is specific to particular pathogens such as NTHi, which is able to interact with Lp(a) in a lysine-dependent manner.

The fibrinolytic system can be exploited by infectious agents for host invasion. Many invasive agents, including bacteria, fungi, and parasites, as well as tumor cells, have been documented to take advantage of Plg [28]. Infectious agents have evolved Plg-binding proteins to participate in Plg capture. Many Plg-binding proteins in bacteria, for instance, *α*-enolase and glyceraldehyde-3-phosphate dehydrogenase (GAPDH), have a common binding mechanism; they interact with LBS within the kringle domains of Plg [15]. Both these proteins are key metabolic enzymes, and their expression is highly conserved across pathogens [29]. Previous studies in our laboratory indicated that recombinant GAPDH and surface enolase of group A streptococci [30], as well as recombinant *α*-enolase of *Staphylococcus aureus* [31], can interact with Lp(a) in a lysine-dependent manner. Moreover, recombinant dihydrolipoamide dehydrogenase of *Pseudomonas aeruginosa* [32] and inosine 5′-monophosphate dehydrogenase of *Staphylococcus aureus* [33] had earlier been shown to bind to Lp(a) through interactions analogous to those of *α*-enolase and GAPDH. Given that the interaction between Lp(a) and NTHi is mainly mediated by LBS, it was reasonable to speculate opsonization by Lp(a) to be possibly applicable to other infectious agents binding to LBS. Structural homology with Plg suggested the potential role of Lp(a) as an opsonin. Because the fibrinolytic system can be hijacked during invasion by infectious agents, the anti-infective activity of Lp(a) with other agents would be worth exploring.

The class B scavenger receptor CD36, which is present on the surface of many cells, including monocytes and macrophages, is a multifunctional receptor that participates in various biological processes [34]. Recognition and internalization of ox-LDL by macrophages are primarily mediated by the CD36 receptor [35]. Lp(a) is known to be sensitive to oxidative modifications [36, 37], thereby enabling its recognition by a scavenger receptor through the apoB100 component [38]. Results of the present study indicated that anti-CD36 mAb inhibited the Lp(a)-induced increase in phagocytosis. Hence, Lp(a) is likely to interact with the scavenger receptor CD36 through its ox-LDL/apoB100 ligand, which may become oxidized during phagocytosis. The results of phagocytosis by U937 monocytes may also indicate the same analysis. We conducted a phagocytosis assay, as previously described [39, 40], using U937 monocytes infected with NTHi at different MOIs. Lp(a), at concentrations of 0.1 *μ*g/mL to 100 *μ*g/mL, failed to increase phagocytosis of NTHi by U937 monocytes after 15, 30, and 60 min incubations (data not shown). Therefore, the ox-LDL/apoB100 ligand on ox-Lp(a) particles may serve as a binding site for the CD36 receptor.

The main limitation of this work was the lack of a validated negative control for NTHi to illustrate the mediation role of Lp(a). Because more than one Plg/Lp(a) binding protein is present in NTHi and not all of these are known, deleting any one of these proteins would not offer an appropriate control to analyze the effects of Lp(a) binding. In addition, although numerous bacteria react with Lp(a) in vitro, many of them also have an affinity for LDL, thus making it difficult to completely isolate the opsonic function of Lp(a). The use of a single donor of Lp(a) and PBMC, although maintaining continuity and stability of the study, is also the obvious limitation of the work. Therefore, research on Lp(a) opsonization should be more thorough in the future.

In conclusion, to the best of our knowledge, we have shown for the first time that Lp(a) can act as an opsonin to increase macrophage phagocytosis of NTHi, which binds to Lp(a) in a lysine-dependent manner, and that CD36, a scavenger receptor on the surface of macrophages, is involved in Lp(a)-mediated opsonization. It is important to gain a better understanding of the relative contribution made by Lp(a) to immune responses, as well as the mechanisms that underlie this function. Further studies are also recommended to understand the role of Lp(a) in immune-related pathophysiological processes.

## Data Availability

The data used to support the findings of this study are available from the corresponding author upon request.

## Abbreviations

apo(a): Apolipoprotein(a)
apoB100: Apolipoprotein B-100
BHI: Brain heart infusion
CFUs: Colony-forming units
CF: Cystic fibrosis
COPD: Chronic obstructive pulmonary disease
DAPI: 4′,6-Diamidino-2-phenylindole
*E. coli*: *Escherichia coli*
ECM: Extracellular matrix
EACA: *ε*-Aminocaproic acid
GAPDH: Glyceraldehyde-3-phosphate dehydrogenase
HMDMs: Human monocyte-derived macrophages
HRP: Horseradish peroxidase
KIV: Kringle IV
KV: Kringle V
LB: Luria-Bertani
LBS: Lysine-binding site
LDL: Low-density lipoprotein
Lp(a): Lipoprotein(a)
NTHi: Nontypeable *Haemophilus influenzae*
PBMC: Peripheral blood mononuclear cell
PE: Protein E
PF: Protein F
Plg: Plasminogen
RFI: Relative fluorescence intensity
rKIV_10_: Recombinant kringle IV type 10
SD: Standard deviation
sBHI: BHI supplemented with hemin and NAD.

## Additional Points

*Summary Sentence*. Lp(a) enhances NTHi phagocytosis by U937 and human monocyte-derived macrophages.
